# Comparison of the Effects of Isoflurane and Propofol as Anesthesia Maintenance on Plasma Mitochondrial DNA Levels in Posterior Spinal Fusion Surgeries

**DOI:** 10.5812/aapm-161767

**Published:** 2025-06-02

**Authors:** Faranak Behnaz, Mehrak Erfanian, Azita Chegini

**Affiliations:** 1 Clinical Research Development Unit of Shohada-e Tajrish Hospital, Shahid Beheshti University of Medical Sciences, Tehran, Iran; 2 Blood Transfusion Research Center, High Institute for Research and Education in Transfusion Medicine, Tehran, Iran

**Keywords:** Mitochondrial DNA, Isoflurane, Propofol, Spinal Fusions, Maintenance of Anesthesia

## Abstract

**Background:**

Tissue injury resulting from surgical procedures leads to the release of various inflammatory agents, such as mitochondrial DNA (mt-DNA). This can trigger inflammatory mechanisms that may harm different organs.

**Objectives:**

In this study, we investigated the effects of isoflurane and propofol on mt-DNA levels during posterior spinal fusion (PSF) surgery.

**Methods:**

After meeting the inclusion criteria, 40 patients scheduled for PSF surgery were enrolled in a prospective randomized controlled clinical trial and randomly divided into groups receiving propofol or isoflurane for maintenance of anesthesia. Mitochondrial DNA levels were measured before surgery, one hour after induction of anesthesia, in the recovery unit, and 24 hours post-surgery.

**Results:**

There was no statistically significant difference between groups regarding age, gender, and mt-DNA levels prior to surgery (P-value > 0.05). However, mt-DNA levels were significantly higher in the isoflurane group one hour after induction of anesthesia (P-value = 0.001), in the recovery unit (P-value = 0.042), and 24 hours after surgery (P-value = 0.018).

**Conclusions:**

Propofol was superior to isoflurane, as demonstrated by a lesser elevation in plasma levels of mt-DNA in PSF patients.

## 1. Background

Choosing the best anesthesia regimen for a surgical procedure is one of the most challenging concerns of any anesthesiologist (1). Postoperative pre-inflammatory stimulation can induce a systemic inflammatory response, potentially leading to dysfunction in multiple organs (2). Major surgeries trigger various inflammatory responses and catecholamine releases, especially norepinephrine (3, 4), which can lead to systemic hypertension, platelet aggregation, tachycardia, increased myocardial demand, impaired wound healing, and impaired blood flow in coronary and pulmonary arteries (5). Early post-surgery inflammatory responses result in an inadequate postoperative course, increased morbidity rates, and early mortality (6). As a result, the primary goal of ideal anesthesia is to utilize the best anesthetics to ensure adequate muscle relaxation, analgesia, and hemodynamic stabilization (7-9).

In the last decade, mitochondria have gained considerable attention due to their significant role in energy production, protein synthesis, and programmed cellular death, with their signaling in critical situations evaluated in multiple studies (10, 11). Based on recent studies, mitochondrial deoxyribonucleic acid (mt-DNA) plays a crucial role in patients' postoperative prognosis by influencing the immune system (12). The mt-DNA is released from human cells in response to stress and critical situations (like surgery), and each mitochondrion possesses different copies of mt-DNA, which are related to the size and quantity of mitochondria (13). This variation reflects the function of these organelles in protein and energy synthesis (14). Factors such as the type of surgical procedure, the duration of anesthesia, and the anesthetics used will influence the amount of mt-DNA released into the plasma (12). Free oxidative radicals will damage mt-DNA, leading to mitochondrial dysfunction, systemic inflammation, and apoptosis (15). Several studies have demonstrated higher levels of mt-DNA in traumatic and surgical circumstances (16, 17).

Considering the increasing prevalence of posterior spinal fusion (PSF) surgeries (18) and limited comparative data on anesthetic effects beyond pain and hemodynamics (19, 20), this study compared the impact of propofol and isoflurane anesthesia on mt-DNA gene levels during and after PSF.

## 2. Objectives

The study aimed to assess the differential effects of these anesthetics on mt-DNA levels in the PSF setting.

## 3. Methods

In a prospective randomized controlled clinical trial, patients scheduled for elective PSF surgery at Shohada Tajrish Hospital in Tehran, Iran, from September 2023 to March 2024, classified as class I or II according to the American Society of Anesthesiologists (ASA) classification, aged between 30 and 70 years, were enrolled in the study (inclusion criteria). Patients with a history of prior PSF, ASA classification of III or IV, a history of malignancies, cardiovascular or chronic inflammatory diseases, long-term corticosteroid use, cases requiring emergent surgeries, a history of substance abuse, and those with intraoperative hemodynamic instability were excluded from the study (exclusion criteria). The study was originally registered by the Shahid Beheshti Committee of Ethics (IR.SBMU.MSP.REC.1402.486) and the Iranian Registry of Clinical Trials (IRCT20190121042444N5).

Considering a significance level of 0.05 and a power of 80%, the sample size was calculated using the Cochrane formula (21). Approximately, considering a 5% margin of error and a potential dropout rate of 10%, 23 participants per group were required. However, due to resource limitations and feasibility considerations, we enrolled 20 participants per group (total n = 40). After detailing the necessary study information for each patient and addressing their related questions, written informed consent was obtained from each individual, and each patient was assigned a random number by the researcher. Patients were randomly divided into two groups (receiving propofol or isoflurane as maintenance of anesthesia) based on their random number utilizing computer software.

Patients' demographic characteristics, including age and gender, were recorded in prepared questionnaires the night before anesthesia. Preoperative routine fasting time for all participants was considered. Before induction of anesthesia, all patients were monitored using routine anesthesia monitoring, including electrocardiography (ECG), pulse oximetry (SPO_2_), heart rate (HR) monitoring, non-invasive blood pressure (NIBP) monitoring, capnography (EtCO_2_), and Bispectral Index (BIS) monitoring. Induction of anesthesia in all patients was similar and consisted of 0.02 mg/kg intravenous (IV) midazolam, 2 - 4 µg/kg IV fentanyl, 1.5 mg/kg IV lidocaine, 1 - 1.5 mg/kg IV propofol, and 0.2 mg/kg IV Cisatracurium as a muscle relaxant. Following tracheal intubation, radial artery cannulation was performed for monitoring. Anesthesia was maintained by a 0.1 - 0.2 mg/kg/min IV infusion of propofol and a 1 - 2.5% mean alveolar concentration (MAC) of isoflurane in each group, aiming to keep the BIS within the range of 40 - 60. 2 mg of IV Cisatracurium and 50 - 100 µg IV fentanyl were repeated at 45-minute intervals.

Blood samples for plasma mitochondrial DNA (mtDNA) measurement were collected at four time points: Before anesthesia induction, one hour post-induction (pre-incision), post-extubation, and 24 hours post-surgery. Samples for the one-hour post-induction and post-extubation time points were drawn from the radial artery catheter, while pre-induction and 24-hour post-surgery samples were drawn from the cubital vein. All samples were collected in ethylene diamine tetraacetic acid (EDTA) coated tubes (non-vacuum K2EDTA tubes, Hebei Xinle Sci & Tech Co., Ltd) and centrifuged at 1600 rpm for 10 minutes. Plasma samples were frozen at -70°C until evaluation, then thawed to 4°C.

Mitochondrial DNA was extracted using the QIAamp Blood DNA mini kit (Qiagen, Germany), and NADH-dehydrogenase subunit 6 (ND6 gene levels, a mt-DNA -specific gene, were quantified by real-time PCR in duplicate. Real-time PCR with SYBR Green was performed to analyze the ND6 gene, and it was normalized to a housekeeping gene.

SPSS software (version 20) was used for data analysis. Data normality was assessed using the Shapiro-Wilk test. Normally distributed quantitative data are presented as mean ± standard deviation; qualitative data are presented as frequencies and percentages. Quantitative variables were analyzed using independent *t*-tests, while qualitative data were analyzed using Pearson's Chi-square tests. Statistical significance was defined as P < 0.05.

## 4. Results

**Figure 1.**
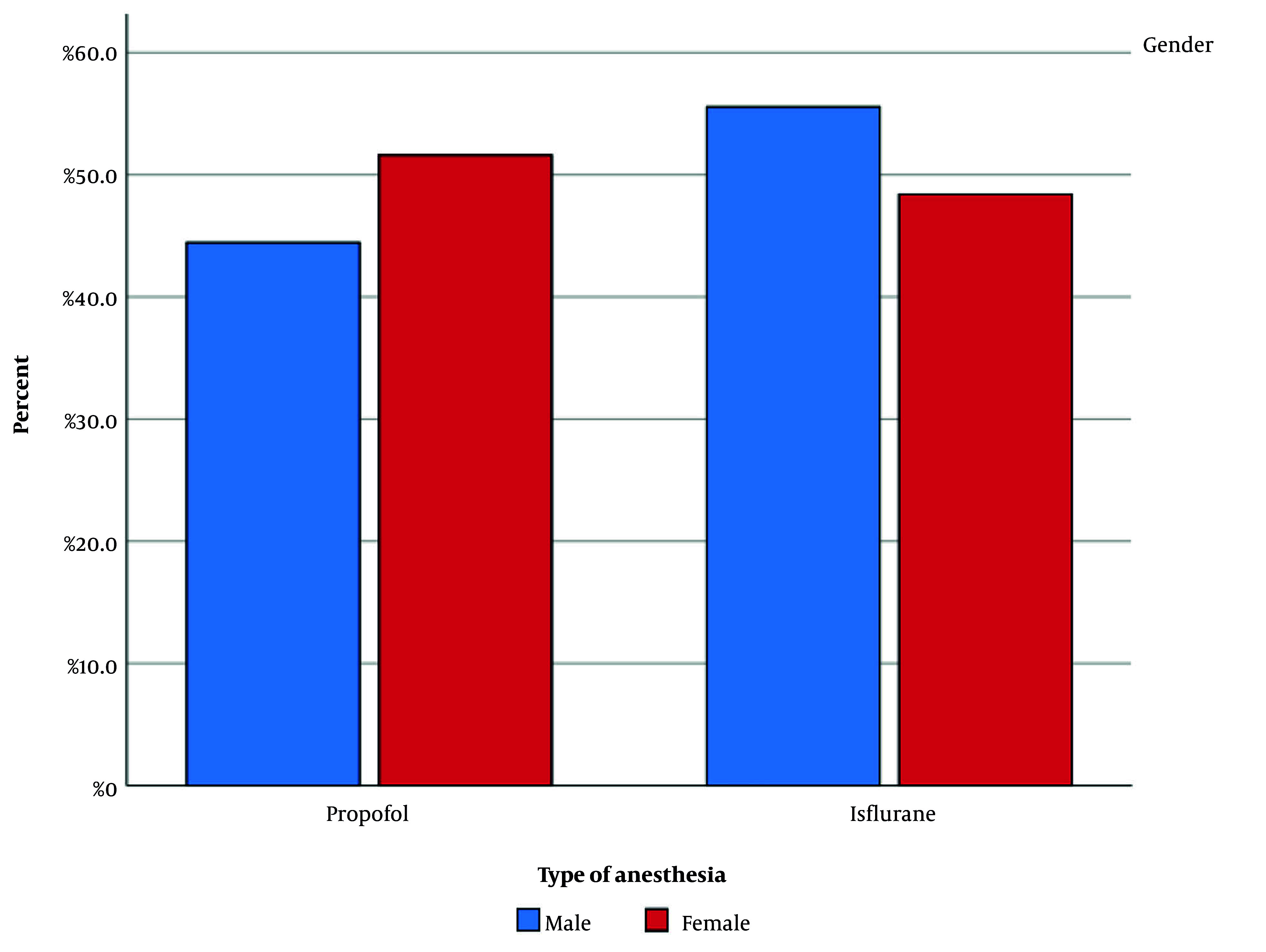
Gender distribution

**Figure 2.**
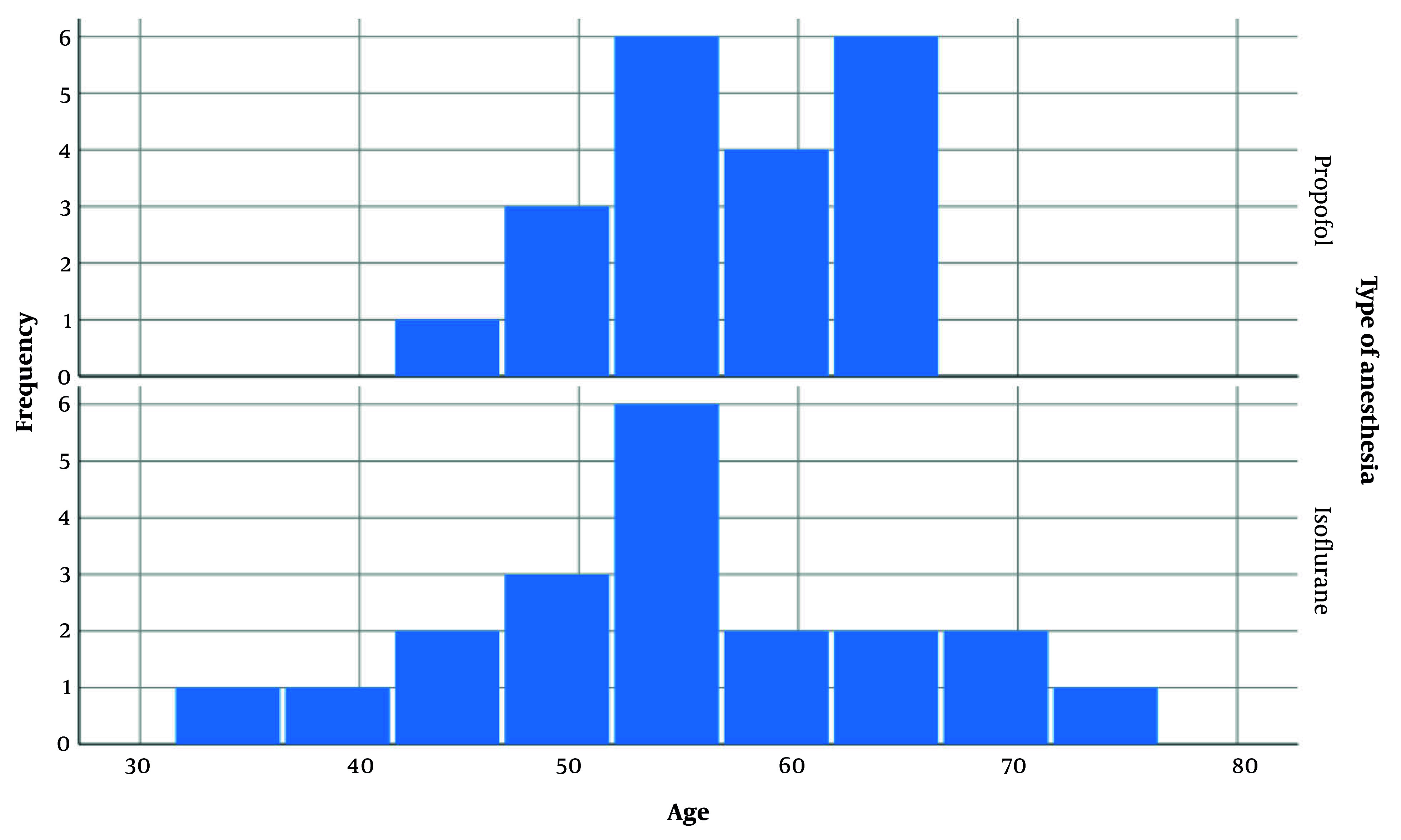
Age distribution

After meeting the inclusion criteria, 40 patients scheduled for elective PSF surgery were enrolled in the study and randomly divided into two groups of 20 patients each. Among the 40 patients, 9 were males and 31 were females. Figure 1 illustrates the gender distribution in both groups. The difference in gender between the groups was evaluated statistically and found to be insignificant (P-value = 0.705). The mean age of patients in the propofol and isoflurane groups was 56.90 ± 5.902 and 54.35 ± 10.282 years, respectively. Figure 2 depicts the age distribution in each group. The difference in patient age between the groups was not statistically significant.

Plasma mtDNA (ND6) levels were measured in patients at four time points: Pre-anesthesia, one hour post-induction, post-extubation (recovery unit), and 24 hours post-surgery. Table 1 shows mean mtDNA plasma levels (ng/μL) for each time point. The two groups did not differ significantly before anesthesia (P = 0.799). Statistically significant differences in mt-DNA levels were observed between groups after anesthesia induction (P = 0.001), in the recovery unit (P = 0.042), and 24 hours post-surgery (P = 0.018), with the propofol group exhibiting lower levels.

**Table 1.** Comparison of Mean of Plasma mt-DNA Levels (ng/μL) Between Patient Groups

Variables	Propofol	Isoflurane	P-Value
**Before anesthesia**	15.30 ± 20.88 × 10^5^	19.68 ± 21.92 × 10^5^	0.799
**After induction**	5.04 ± 6.57 × 10^5^	19.81 ± 17.55 × 10^5^	0.001
**Recovery**	8.38 ± 10.29 × 10^5^	15.13 ± 14.69 × 10^5^	0.042
**24 (h) later**	0.10 ± 0.15 × 10^5^	3.44 ± 6.00 × 10^5^	0.018

Abbreviation: mt-DNA, mitochondrial DNA.

## 5. Discussion

Anesthesia research is evolving beyond hemodynamic and hematologic effects (22) to include molecular, genetic, and pharmacogenetic investigations. Enabled by new molecular technologies, pharmacogenetics is rapidly advancing the understanding of individual variations in drug response based on genetic factors. This progress holds promise for personalized anesthetic regimens to improve patient comfort, safety, and reduce morbidity and mortality (23). Furthermore, studies show that elevated postoperative plasma mt-DNA levels, a marker of cellular injury and inflammation, may negatively impact outcomes (24, 25). This elevation may lead to long-term complications, including sepsis and even death (26, 27). The increase in plasma mt-DNA levels in patients admitted to the intensive care unit (ICU) has resulted in severe respiratory distress and a higher mortality rate (28). In 2014, McIlroy et al. showed the elevation of mt-DNA and inflammatory cytokines in the postoperative period (29). Pencovich et al. in 2021 demonstrated the relationship of elevation in mt-DNA plasma level following pancreaticoduodenectomy (25). All of this evidence shows the crucial role of mt-DNA as a predictive biomarker for postoperative inflammatory response and its side effects.

Propofol is an IV anesthetic first produced in the United Kingdom and introduced into the market in 1986 in Europe and the United States of America (30, 31). Due to its shorter half-life, lack of serious side effects, and low incidence of postoperative nausea and vomiting, propofol has become the most popular anesthetic for induction and maintenance of anesthesia in the last three decades (32-34). Several studies show the anti-inflammatory properties of this agent (35, 36). Isoflurane is a volatile anesthetic and a halogenated ether formula (37, 38). Compared to other volatile anesthetics such as halothane, isoflurane preserves myocardial contractility by more than 20%. Some recent studies have demonstrated the anti-inflammatory effects of this volatile (39). Consistent with previous research, our study found that both propofol and isoflurane reduced mtDNA levels within 24 hours, with propofol causing a significantly larger reduction.

Kotani et al. demonstrated that propofol induces lower inflammatory respiratory responses and pre-inflammatory cytokine expression compared to isoflurane (40). This study reviewed the effect of anesthetics on different surgical procedures. Niezgoda and Morgan in 2013 showed the importance of anesthetic effects on mt-DNA mutations and suggested the preoperative evaluation of mitochondrial gene mutations (41). In 2015, Sayed et al. showed that propofol causes fewer improper inflammatory responses than isoflurane (42). Similar to our study, they measured the inflammatory factors at different intervals. However, mt-DNA plasma level was not the indicator of inflammatory response in Sayed’s study. Kajimoto et al. in 2016 showed a different result, indicating the superiority of isoflurane as maintenance of anesthesia with lower plasma levels of mt-DNA (43). Unlike our study, this was a rodent study. In 2019, Safari et al. compared the anti-inflammatory properties of isoflurane and propofol as the maintenance of anesthesia in brain tumor surgeries. The results showed that isoflurane causes more elevation in plasma levels of inflammatory cytokines (12).

Our study was limited by the evaluation of only one subtype of mt-DNA. Therefore, we suggest conducting studies regarding other subtypes. Another limitation was that our study did not consider other anesthesia components, including surgery duration, amount of blood loss, and levels of surgery. We suggest conducting different studies on other anesthetics in the settings of other major surgeries.

### 5.1. Conclusions

The anti-inflammatory properties of propofol were superior to those of isoflurane, as demonstrated by a lesser elevation in plasma levels of the mt-DNA gene. Propofol might be a preferable anesthetic for maintaining anesthesia during PSF surgery.

## Data Availability

The dataset presented in the study is available on request from the corresponding author during submission or after publication.

## Clinical Trial Registration Code:

IRCT20190121042444N5.
